# *In vitro* virucidal activity of mouthwashes on SARS‐CoV‐2

**DOI:** 10.1111/odi.14205

**Published:** 2022-04-25

**Authors:** Alessio Buonavoglia, Gianvito Lanave, Serena Marchi, Pantaleo Lorusso, Emanuele Montomoli, Vito Martella, Michele Camero, Carlo Prati, Claudia Maria Trombetta

**Affiliations:** ^1^ Department of Biomedical and Neuromotor Sciences Dental School University of Bologna Bologna Italy; ^2^ 9295 Department of Veterinary Medicine University of Bari Valenzano Italy; ^3^ 9313 Department of Molecular and Developmental Medicine University of Siena Siena Italy; ^4^ Department of Emergency and Organ Transplantation Section of Anesthesia and Intensive Care Aldo Moro University of Bari Bari Italy; ^5^ VisMederi srl Siena Italy; ^6^ VisMederi Research srl Siena Italy

**Keywords:** *in vitro*, mouthwashes, SARS‐CoV‐2, variants, virucidal activity

## Abstract

**Objectives:**

The objective of the study was to evaluate the in vitro virucidal activity of commercial mouthwashes against SARS‐CoV‐2 and variants of concern.

**Materials and Methods:**

Antiviral activity was assessed at different time intervals, based on common use of these products by titrating residual viral infectivity on Vero E6 cells.

**Results:**

All the mouthwashes were effective to reduce the infectious titers of SARS‐CoV‐2 and its tested variants. Mouthwashes Listerine^®^ Cool Mint milder taste and Listerine^®^ Cavity Protection milder taste reduced the infectious viral titer by up to 3.9 log10 after 30 s, while mouthwash Cetilsan^®^ Sugar Free was able to reduce the viral titer by 2.2–2.9 log10 at all tested time intervals. Mouthwash Curasept^®^ ADS DNA Intensive treatment was less effective to decrease viral infectivity (0.7–2.2 log10 TCID50/ml at all tested time intervals). Interestingly, the Gamma variant appeared more resistant to treatment in vitro with the different mouthwashes.

**Conclusions:**

In this study, we were able to assess the ability of different mouthwashes to in vitro decrease the infectivity of SARS‐CoV‐2 and its variants, and we observed that Gamma variant of concern was more resistant to treatment with mouthwashes.

## 1 INTRODUCTION

Severe Acute Respiratory Syndrome Coronavirus‐2 (SARS‐CoV‐2), declared pandemic since March 2020 (WHO, 2020), causes the coronavirus disease 2019 (COVID‐19). Coronaviruses belong to a large family of viruses circulating widely among humans and animals (Weiss et al., 2005).

Since the beginning of the pandemic, five SARS‐CoV‐2 variants of concern (VoCs) have emerged, thus posing an increased risk to global public health (Sanyaolu et al., 2021; WHO, 2021).

SARS‐CoV‐2 is transmitted by direct, indirect, or close contact with saliva and respiratory secretions of infected people produced by sneezing, coughing, breathing, and phonating. A high salivary load of SARS‐CoV‐2 in COVID‐19 patients may be responsible for the increasing risk of contamination of the surrounding environment (Aboubakr et al., 2021). As saliva is a potential vehicle for viral spread (Vaz et al., 2020), reducing salivary virus load may help prevent its spread.

Healthcare workers are likely to be at higher risk of severe COVID‐19 infection due to continuous exposure to the saliva of potentially infected patients (Prati et al., 2020). The virus may be transmitted during dental and otolaryngological procedures that produce droplets which can remain suspended in the air from few minutes to hours before laying on surfaces (Mick & Murphy, 2020; Gandolfi et al., 2020; Kohanski et al., 2020; Valk and In ‘t Veen, 2021). Droplets produced by COVID‐19 patients might be inhaled by healthcare professionals (i.e., dentists, hygienists, students, and medical doctors) and may float in the air and land on office surfaces increasing the risk for contamination (Sommerstein et al., 2020; Ueki et al., 2020).

The COVID‐19 pandemic prompted a dramatic closure or limitation of dental services worldwide, thus causing serious health and economic consequences (Marcenes, 2020). The increasing risks in SARS‐CoV‐2 transmission via saliva and respiratory secretions during dental procedures has led to the use of pre‐operating mouthwashes (Tadakamadla et al., 2021; Mendoza et al., 2022; Silva et al., 2021) as additional preventive measures together with physical barriers such as facial masks and facial barriers.

SARS‐CoV‐2 is surrounded by a lipid envelope that includes spike (S) glycoproteins able to interact with cell receptors widely expressed in mucosal tissues, gingiva, tongue, and salivary glands (Carrouel et al., 2021; Hamming et al., 2004). The interference with the lipid envelope is regarded as a virucidal approach to counteract enveloped viruses, including coronaviruses (Siddharta et al., 2017). Mouthwashes employed to lower active virus load in the oropharynx are mainly able to damage or destroy the viral envelope (O'Donnell et al., 2020).

Cetylpyridinium chloride (CPC)‐based mouthwashes have been demonstrated able to induce in vitro virucidal effect against SARS‐CoV‐2 and Alpha variant (Komine et al., 2021; Meyers et al., 2021; Munoz‐Basagoiti et al., 2021). Chlorhexidine digluconate (CHX)‐based mouthwashes were able to inactivate SARS‐CoV‐2 by 42.5 to >99% applying incubation times ranging from 30 s to 10 min (Davies et al., 2021; Jain et al., 2021; Komine et al., 2021; Meister et al., 2020). Sodium lauryl sulfate (SLS)‐based mouthwashes showed promising inhibitory activities against the interaction between the S protein of SARS‐CoV‐2 and cell receptors either in the presence or absence of saliva (Tateyama‐Makino et al., 2021). This suggests the ability of SLS in reducing the activity of the virus and inhibiting the entry point of the virus in the oral cavity.

The virucidal activity of essential oils (EOs)‐based mouthwashes in vitro against SARS‐CoV‐2 was recently reported with a complete inactivation of the virus after 30 s to 2 min of incubation (Cimolai, 2020; Davies et al., 2021; Meister et al., 2020; Meyers et al., 2021).

In this study, we evaluated the in vitro virucidal activity of four commercial mouthwashes against the original Wuhan strain of SARS‐CoV‐2 and the Alpha, Beta, and Gamma VoCs.

## 2 MATERIALS AND METHODS

### 2.1 Mouthwashes (M)

The following commercial mouthwashes (M) were used:

MA: Listerine^®^ Cool Mint milder taste (alcohol‐free mouthwash containing SLSs and EOs Eucalyptol, Thymol, Menthol);

MB: Listerine^®^ Cavity Protection milder taste (alcohol‐free mouthwash containing SLSs and EOs Eucalyptol, Thymol, Menthol and *Camelia Sinensis* leaf extract, and caffeine);

MC: Cetilsan^®^ Sugar Free (mouthwash containing CPC 0.1%, alcohol and EOs eucalyptol, eugenol);

MD: Curasept^®^ ADS DNA Intensive treatment (alcohol‐free mouthwash containing CHX 0.2%, polyvinylpyrrolidone/vinyl acetate copolymer, Sodium DNA, EOs peppermint M644, anethole, menthol, green mint, cloves, and cinnamon).

### 2.2 Viruses and cell cultures

The virucidal activity was evaluated against the wild type (wt) SARS‐CoV‐2 (2019‐nCov/Italy‐INMI1 strain, clade V strain, Wuhan strain) and the Alpha, Beta, and Gamma VoCs. The wt, Alpha, and Beta strains were purchased from the European Virus Archive goes Global (EVAg) while the Gamma strain was kindly provided by the University of Siena, Department of Medical Biotechnology.

Vero E6 cells (ATCC ‐ CRL 1586) were cultured in Dulbecco's modified Eagle's medium (DMEM)—High Glucose (Euroclone, Pero, Italy) supplemented with 2 mM L‐ Glutamine (Lonza,), 100 units/ml penicillin‐streptomycin mixture (Lonza) and 10% of fetal bovine serum (FBS) (Euroclone), in a 37°C, 5% CO_2_ humidified incubator.

Cells were seeded in T175 cm^2^ flask at a density of 1 × 10^6^cells/ml. After 18–20 h, the sub‐confluent cell monolayer was infected with 3.5 ml of DMEM 2% FBS containing the SARS‐CoV‐2 strains at a multiplicity of infection (MOI) of 0.01. After 1 h of incubation at 37°C in a humidified atmosphere with 5% CO_2_, 50 mL of DMEM containing 2% FBS were added. The flasks were observed daily, and the viruses were harvested when 80%–90% of the cells manifested cytopathic effect (CPE).

Each virus was titrated in serial 1 log dilutions (from 1 log to 11 log) to obtain a 50% tissue culture infective dose (TCID50) on 96‐well plate of VERO E6 cells.

The viral titers of stock viruses used to assess virucidal activity were 10^6.41^/ml tissue culture infectious doses (TCID_50_/ml) for the wt virus, 10^6.33^TCID_50_/ml for Alpha, 10^6.33^TCID_50_/ml for Beta, and 10^5.67^ TCID_50_/ml for Gamma VoCs.

### 2.3 Virucidal activity assay

The potential virucidal activity of mouthwashes against SARS‐CoV‐2 and its VoCs was assessed by pre‐treatment of the viruses with mouthwashes A to D. In detail, the viral stock of each virus was put in contact with the same amount of each mouthwash. After 30 s (T1), 1 min (T2), and 3 min (T3) of incubation at room temperature, the samples were diluted tenfold from 10^−1^ to 10^−11^ in DMEM and subjected to viral titration in Vero E6 cells. For each virus, a virus control (VC) was prepared by titrating the stock virus, as reported in the previous paragraph.

### 2.4 Viral titration

One hundred µl of each virus‐mouthwash mixture dilution (8 replicates for each dilution) or virus were then added to a 96‐well plate containing an 80% confluent Vero E6 cell monolayer. The plates were incubated for 72 h for wt virus and 96 h for the VoCs at 37°C, 5% CO_2_ in humidified atmosphere, and checked for presence/absence of CPE by an inverted optical microscope. A CPE higher than 50% of the monolayer indicated viral infection. Based on the CPE, the viral titer was calculated using the Reed‐Muench method (Reed et al., 1938). All the experiments were performed in triplicate.

### 2.5 Data analysis

Normality of distribution was assessed by Shapiro–Wilk test. Data from virucidal activity of mouthwashes were expressed as mean ± standard deviation (SD) and analyzed by analysis of variance (ANOVA) using the Tukey test as post hoc test (statistical significance set at 0.05). Statistical analyses were performed with the software GraphPad Prism v.8.0.0 (GraphPad Software,).

## 3 RESULTS

Preliminary evaluation of mouthwashes (without SARS‐CoV‐2) on Vero E6 cells revealed evidence of cytotoxic effects in undiluted solutions and, occasionally, in the wells containing the 10^−1^ and 10^−2^ dilution of the mouthwashes/virus mixture.

The results of viral titrations on Vero E6 cells at T1, T2, and T3 contacts of the control virus and of the mouthwashes with the wt virus and the Alpha, Beta, and Gamma VoCs are reported in Table 1. Control viruses did not show significant variations in the viral titers at different time intervals. Moreover, the virucidal activity of the mouthwashes against wt and VoCs was statistically compared with VC and reported in Table 2.

**TABLE 1:** In vitro evaluation of the virucidal activity of mouthwashes (A, B, C, D) against SARS‐CoV‐2 and variants (Alfa, Beta, and Gamma) after time contact of 30 s (T1), 1 min (T2), and 3 min (T3)

		SARS‐CoV−2				Alpha variant				Beta variant				Gamma variant			
M	Main component	Titer of virus +mouthwashes			cv	Titer of virus +mouthwashes			cv	Titer of virus +mouthwashes			cv	Titer of virus +mouthwashes			cv
		T1	T2	T3		T1	T2	T3		T1	T2	T3		T1	T2	T3	
MA	SLS and EOs	2.9* (3.5) ^∆^	2.5 (3.9)	2.5 (3.9)	6.4	2.9 (3.4)	2.5 (3.8)	2.5 (3.8)	6.3	2.8 (3.5)	2.5 (3.8)	2.5 (3.8)	6.3	2.9 (2.8)	2.5 (3.2)	2.5 (3.2)	5.7
MB	SLS, EOs and caffeine	2.6 (3.8)	2.5 (3.9)	2.5 (3.9)	6.4	2.5 (3.8)	2.5 (3.8)	2.5 (3.8)	6.3	2.5 (3.8)	2.5 (3.8)	2.5 (3.8)	6.3	3.1 (2.6)	2.5 (3.2)	2.5 (3.2)	5.7
MC	CPC 0.1%	3.5 (2.9)	3.5 (2.9)	3.5 (2.9)	6.4	3.5 (2.8)	3.5 (2.8)	3.5 (2.8)	6.3	3.5 (2.8)	3.5 (2.8)	3.5 (2.8)	6.3	3.5 (2.2)	3.5 (2.2)	3.5 (2.2)	5.7
MD	CHX 0.2% and EOs	5.4 (1)	5.3 (1.1)	4.2 (2.2)	6.4	5.4 (0.9)	5.2 (1.1)	5.2 (1.1)	6.3	5.6 (0.7)	5.3 (1)	5 (1.3)	6.3	4.8 (0.9)	4.9 (0.8)	4.7 (1)	5.7

Abbreviations: *, Viral titer expressed as log 10 TCID_50_/ml; ∆, log10 TCID_50_/ml reduction in viral titer of virus +mouthwashes compared to virus control; CHX, chlorhexidine; CPC, cetylpyridinium; cv, control virus; EOs, essential oils; M, mouthwash; SLS, sodium lauryl sulfate.

**TABLE 2:** Virucidal activity of mouthwashes (MA, MB, MC MD) against SARS‐CoV‐2 and variants (Alpha, Beta, Gamma) after time contact of 30 s (T1), 1 min (T2), and 3 min (T3) compared with the respective control virus (CV)

Comparisons	Viral titers (log10 TCID_50_/50µl)								
	T1			T2			T3		
	MDV	95% CI	*p* Value	MDV	95% CI	*p* Value	MDV	95% CI	*p* Value
SARS‐CoV−2 cv vs MA+v	3.5	[2.84; 4.16]	<0.001***	3.9	[3.26; 4.54]	<0.001***	3.9	[3.26; 4.54]	<0.001***
SARS‐CoV−2 cv vs MB+v	3.8	[3.14; 4.46]	<0.001***	3.9	[3.26; 4.54]	<0.001***	3.9	[3.26; 4.54]	<0.001***
SARS‐CoV−2 cv vs MC+v	2.9	[2.24; 3.56]	<0.001***	2.9	[2.26; 3.54]	<0.001***	2.9	[2.26; 3.54]	<0.001***
SARS‐CoV−2 cv vs MD+v	1.0	[0.34; 1.66]	0.005**	1.1	[0.46; 1.74]	0.002**	2.2	[1.62; 2.91]	<0.001***
Alpha cv vs MA+v	3.4	[2.76; 4.04]	<0.001***	3.8	[3.25; 4.35]	<0.001***	3.8	[3.25; 4.35]	<0.001***
Alpha cv vs MB+v	3.8	[3.16; 4.44]	<0.001***	3.8	[3.25; 4.35]	<0.001***	3.8	[3.25; 4.35]	<0.001***
Alpha cv vs MC+v	2.8	[2.16; 3.44]	<0.001***	2.8	[2.25; 3.34]	<0.001***	2.8	[2.25; 3.34]	<0.001***
Alpha cv vs MD+v	0.9	[0.26; 1.54]	0.007**	1.1	[0.55; 1.65]	<0.001***	1.1	[0.55; 1.65]	<0.001***
Beta cv vs MA+v	3.5	[2.98; 4.02]	<0.001***	3.8	[3.21; 4.39]	<0.001***	3.8	[3.14; 4.46]	<0.001***
Beta cv vs MB+v	3.8	[3.28; 4.32]	<0.001***	3.8	[3.21; 4.39]	<0.001***	3.8	[3.14; 4.46]	<0.001***
Beta cv vs MC+v	2.8	[2.28; 3.32]	<0.001***	2.8	[2.21; 3.39]	<0.001***	2.8	[2.14; 3.46]	<0.001***
Beta cv vs MD+v	0.9	[0.38; 1.42]	0.002**	1.0	[0.40; 1.59]	0.002**	1.3	[0.64; 1.96]	<0.001***
Gamma cv vs MA+v	2.8	[2.30; 3.30]	<0.001***	3.2	[2.65; 3.75]	<0.001***	3.2	[2.54; 3.86]	<0.001***
Gamma cv vs MB+v	2.6	[2.10; 3.10]	<0.001***	3.2	[2.65; 3.75]	<0.001***	3.2	[2.54; 3.86]	<0.001***
Gamma cv vs MC+v	2.2	[1.70; 2.70]	<0.001***	2.2	[1.65; 2.75]	<0.001***	2.2	[1.54; 2.86]	<0.001***
Gamma cv vs MD+v	0.7	[0.20; 1.20]	0.007**	0.8	[0.25; 1.35]	0.006**	1.0	[0.34; 1.66]	0.004**

Abbreviations: 95% CI, 95% confidence interval; M, mouthwash; MDV, mean difference of viral titers expressed as log10TCID_50_/ml; v, virus.

**Very significant.

***Highly significant.

All mouthwashes significantly (*p* < 0.05) reduced viral titers of wt virus and VoCs as compared to VC in the different time intervals evaluated in this study (Table 2). MA and MB significantly decreased viral titers of wt virus, Alpha, and Beta VoCs by 3.4–3.9 log10 TCID50/ml (*p* < 0.001) at all the time points while the viral titers of the Gamma VoC were reduced by 2.6–2.8 log10 TCID50/ml (*p* < 0.001) after 30 s and by 3.2 log10 TCID50/ml (*p* < 0.001) after 1 min and 3 min time contacts. MC was able to significantly reduce the viral titers of wt virus, Alpha, and Beta VoCs by 2.8 to 2.9 log10 TCID50/ml (*p* < 0.001) at all time intervals while MC decreased Gamma VoC by 2.2 log10 TCID50/ml (*p* < 0.001) at all time intervals.

MD moderately decreased, yet significantly, the infectious titer of SARS‐CoV‐2 wt and of the VoCs by 0.7–1.3 log10 TCID50/ml (from *p* = 0.007 to *p* < 0.001) over all time intervals, with exception of the wt virus that lost 2.2 log10 TCID50/ml (*p* < 0.001) after 3 minutes.

## 4 DISCUSSION

Before SARS‐CoV‐2 pandemic, the development of mouthwashes was essentially aimed at the reduction of the bacterial load in the oral cavity and included products to release Fluoride as caries prevention, and products to reduce periodontal pathogen bacteria. Moreover, the efficacy of mouthwashes against viruses has been reported (Carrouel et al., 2021; Eggers et al., 2018; Shewale et al., 2021). However, since 2020, the virucidal activity of mouthwashes against SARS‐CoV‐2 has been investigated in detail, as these products, if opportunely conceived, could offer a useful tool to reduce the risk of infection.

The commercial availability of pre‐procedural mouthwashes with specific antiviral properties against SARS‐CoV‐2 is regarded as a safe device for medical and dental practitioners. To date, CPC or iodine‐povidone (PVP‐I)‐based mouthwashes are the most recommended to reduce SARS‐CoV‐2 viral load in droplets and aerosols generated during dental procedures (Herrera et al., 2020; Seneviratne et al., 2021; Shet et al., 2022; Xu et al., 2021). However, the use of PVP‐I is contraindicated in patients with allergy to iodine, with thyroid disease, pregnancy, or treatment with radioactive iodine (Gray et al., 2013). Hydrogen peroxide‐, CHX‐, cyclodextrin‐, Citrox‐, EO‐, and SLS‐ based mouthwashes tested also effective against SARS‐CoV‐2. Several proprietary mouthwash formulations contain alcohol (ethanol), and in some products, the concentration of ethanol can be as high as 26% (Huang et al., 2021). Moreover, in a recent review data regarding the efficacy of experimental mouthwashes against SARS‐CoV‐2 have been reported (Chen & Chang, 2021). Also, there is evidence that VoCs may differ in terms of biological properties (antigenicity, fitness, and transmission velocity) and therefore assessing the virucidal activity of mouthwashes with different VOCs could be helpful to obtain a more precise picture.

In this study, we tested the in vitro virucidal activity of SLS‐, CPC‐, and CHX‐based commercial mouthwashes against wt SARS‐CoV‐2 and three VoCs. The virucidal effects were evaluated at different contact times (30 s, 1 min, and 3 min) which are the common time intervals used for mouthwash application (Jenkins et al., 1994).

SLS‐ and CPC‐based mouthwashes showed highly significant virucidal activity against all the SARS‐CoV‐2 strains tested in this study after as few as 30 s of contact. These results mirror what observed in similar in vitro studies using a feline coronavirus strain (Buonavoglia et al., 2021).

In this study, the virucidal activity of mouthwashes in their commercial formulations has been tested. We cannot exclude that over the main components other minor ingredients or excipients may have exerted virucidal activity alone or in synergy. For example, thymol may have antiviral effect and has been suggested as general environmental disinfectant (Kowalczyk et al., 2020).

Among the antiseptics, according to the available scientific literature, CPC offers the most encouraging results in vitro, tested with SARS‐CoV‐2 wt and with Alpha VoC (Munoz‐Basagoiti et al., 2021). In our study, the CPC‐based mouthwash MC used at a concentration of 0.1% was able to reduce the viral titer of wt virus and of VoCs by up to 2.9 log 10 at all the time intervals. The antiviral effect of CPC against coronaviruses is probably based on its lysosomotropic activity and ability to destroy viral capsids (Baker et al., 2020).

Although the efficacy of CHX against SARS‐CoV‐2 has been demonstrated (Jain et al., 2021), the virucidal activity of CHX‐based mouthwashes is considered controversial (Herrera et al., 2020). The 5th edition of the guidelines for the diagnosis and treatment of SARS‐CoV‐2 pneumonia released by the National Health Commission of the People's Republic of China (Chinese Centre for Disease Control & Prevention, 2020) indicates that CHX‐mouthwashes, used in dental practices, are not effective in reducing the viral load of COVID‐19 (Cavalcante‐Leao et al., 2021; Peng et al., 2020). This finding mirrors the limited effects against SARS‐CoV‐2 strains observed in our study.

Interestingly, we observed a lower virucidal activity for all the tested mouthwashes against the Gamma VoC. The reason for this unique resistance pattern is not clear and could be related to increased virus stability or to increased tenacity of the receptorial interaction or to number of mutations of the S protein and genomic alterations in comparison with other variants (Mohammadi et al., 2021). Interestingly, this phenomenon could account, in part, for the increased speed of transmission of this variant in the human population and should be investigated more in depth, considering the relevant implications. A limit of this study was the fact that we did not confirm the results in vivo, for instance evaluating the viral titer in the saliva of SARS‐CoV‐2‐infected patients before and after mouth rinsing with mouthwashes. Regardless, these findings indicate that different VoCs of SARS‐CoV‐2 should be used when evaluating products used for virus inactivation.

The translational value of our results to the clinical use should be assessed more precisely, since some factors, not considered in vitro, could negatively impact on the antiviral efficacy of mouthwashes in the oral cavity. This could include the contact times, the volume, and palatability of the mouthwash. Also, since the rinsing and virucidal effect of mouthwashes is limited to the oral cavity, this would not have any effect on the virus shed by the nasal route.

## 5 CONCLUSIONS

In conclusion, we were able to assess the in vitro ability of different mouthwashes to decrease the infectivity of SARS‐CoV‐2 and its variants, and we observed that Gamma VoC was apparently more resistant to treatment with the tested mouthwashes.

## ACKNOWLEDGEMENTS

Open Access Funding provided by Università degli Studi di Siena within the CRUI‐CARE Agreement.

## CONFLICT OF INTEREST

The authors declare that they have no conflict of interest.

## AUTHOR CONTRIBUTIONs

**Alessio Buonavoglia:** Conceptualization; Writing – original draft. **Gianvito Lanave:** Data curation; Formal analysis; Visualization; Writing – review & editing. **Serena Marchi:** Formal analysis; Methodology; Writing – review & editing. **Pantaleo Lorusso:** Visualization; Writing – review & editing. **Emanuele Montomoli:** Resources; Writing – review & editing. **Vito Martella:** Visualization; Writing – review & editing. **Michele Camero:** Formal analysis; Writing – review & editing. **Carlo Prati:** Visualization; Writing – review & editing. **Claudia Maria Trombetta:** Formal analysis; Methodology; Writing – review & editing.

## PEER REVIEW

The peer review history for this article is available at https://publons.com/publon/10.1111/odi.14205.

## Data Availability

The data sets used and/or analyzed during the current study are available from the corresponding author on reasonable request.
